# Concomitant Urothelial Cancer and Renal Tuberculosis

**DOI:** 10.1155/2014/625153

**Published:** 2014-07-14

**Authors:** Sheray N. Chin, Tanya Foster, Gurendra Char, Audene Garrison

**Affiliations:** ^1^Department of Pathology, University Hospital of the West Indies, University of the West Indies, Mona, Kingston 7, Jamaica; ^2^Department of Medicine, University Hospital of the West Indies, University of the West Indies, Mona, Kingston 7, Jamaica

## Abstract

We report a case of coexisting urothelial cancer and renal tuberculosis in the same kidney. The patient is a 72-year-old female with a remote history of treated pulmonary tuberculosis who presented with haematuria, initial investigation of which elucidated no definitive cause. Almost 1 year later, a diagnosis of metastatic urinary tract cancer was made. The patient received chemotherapy for advanced collecting duct type renal cell carcinoma, based on histological features of renal biopsy. Subsequent confirmatory immunostains however led to a revised diagnosis of urothelial cancer, necessitating a change in chemotherapy regimen. A diagnosis of ipsilateral renal tuberculosis was made based on TB-PCR testing of renal biopsy tissue and anti-TB therapy was coadministered with chemotherapy. The patient died 9 months after diagnosis of metastatic urothelial cancer.

## 1. Introduction

The occurrence of tuberculosis and urothelial cancer in the same kidney is rare. To our knowledge, only one previous case has been reported in the English language literature [1]. We report the case of a patient investigated for haematuria and found to have coexisting renal tuberculosis and urothelial cancer.

## 2. Case Report

A 72-year-old Chinese woman presented with recent onset gross painless haematuria, with no associated genitourinary or constitutional symptoms and no history of bleeding disorders. Medical comorbidities included long-standing diabetes mellitus and hypertension. In addition, she gave a history of pulmonary tuberculosis (TB) diagnosed over 30 years ago, which was completely treated with no sequelae. The diagnosis of TB was made while living in Jamaica after emigrating from China many years earlier.

**Figure 1:**
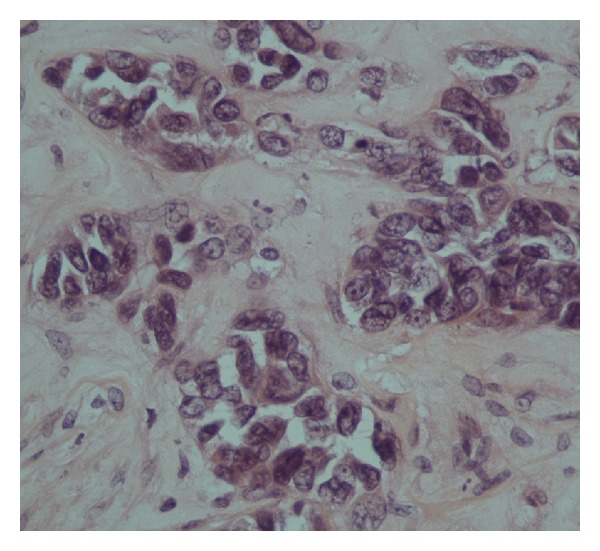
Photomicrograph of renal cell adenocarcinoma.

Investigations to elucidate a cause for haematuria included MDCT urogram, cystoscopy, and urine cytology, which revealed no abnormalities. Urine cultures for tuberculosis were negative. There was spontaneous resolution of haematuria without any specific therapy. Eight months later, however, the patient developed constitutional symptoms with significant weight loss, anorexia and lethargy, and associated severe lower back pain. Magnetic resonance imaging (MRI) scan of the lumbosacral spine was performed and it demonstrated marked infiltration of L1 and L2 vertebral bodies, possibly due to metastatic infiltration. Computed tomography (CT) imaging of the chest, abdomen, and pelvis demonstrated hepatic metastases, an enlarged right kidney with lower pole nodule, and calcified apical lung nodules. A right renal tumor was suspected, and renal biopsy was performed which was examined in toto and at multiple levels. It was comprised of cores of renal tissue containing glomeruli and tubules within fibrous stroma. The tubules were lined by cuboidal to short columnar epithelial cells exhibiting marked hyperchromasia and prominent pleomorphism with bizarre tumor giant cell formation (Figure 1). The overall histological picture was that of a renal cell carcinoma, collecting duct type. Confirmatory immunostains were not immediately available. No granulomata were seen; however, based on the history of primary TB, biopsy tissue was submitted for tuberculosis-polymerase chain reaction (TB-PCR) testing.

A diagnosis of metastatic collecting duct carcinoma was made. Given the patient's progressive symptoms, chemotherapy (carboplatin/paclitaxel) with palliative intent was commenced. She also received palliative external beam radiation therapy to the L1 vertebra, with improvement in back pain. After starting chemotherapy, results of TB-PCR testing became available. Mycobacterium tuberculosis complex was identified using DNA-sequencing of PCR products from two of four urine specimens and two of two aspirates from the right kidney. Based on the clinical history and the positive PCR results, anti-TB therapy with RIPE (rifampicin, isoniazid, pyrazinamide, and ethambutol) was instituted pending drug sensitivity testing results. The patient experienced clinical palliative benefit, with improvement in back pain and improved appetite with weight gain. In view of the dual pathology and concurrent treatments it was difficult to ascertain if this clinical response was due primarily to cancer chemotherapy, antituberculosis therapy, or both.

Radiological assessment for response after 3 cycles of chemotherapy showed progression of both hepatic and bony metastases. By that time, pathology review with adjunctive immunostaining tests became available. Tumor cells stained positive for p63, high molecular weight cytokeratin, and CK7 and stained negative for CD10, racemase, PAX-8, and vimentin. The overall features were most consistent with high-grade urothelial carcinoma. With evidence of radiological progression and the potential for response of urothelial malignancies to cisplatin-based regimens, the chemotherapy regimen was changed to gemcitabine/cisplatin. Despite this change, radiological progression was confirmed on CT abdomen after 3 cycles of gemcitabine/cisplatin. The patient had clinical progression with deterioration in performance status and chemotherapy was discontinued. Best supportive care was initiated and she died approximately 9 months after the initial diagnosis of metastatic cancer.

## 3. Discussion

Renal tuberculosis is a well-known complication of pulmonary TB with an 8–10% risk of genitourinary TB developing in a patient with known pulmonary tuberculosis [2]. Urothelial carcinoma of the renal pelvis accounts for 7% of renal tumors [3]. The occurrence of TB and urothelial cancer in the same individual is rare. Feeney et al. estimated that the likelihood of these diseases occurring simultaneously in an individual is approximately 1 in 10 billion [1]; occurrence in the same kidney would be even more rare.

Tuberculosis has been reported to complicate a myriad of cancers [4], with the highest prevalence noted for lung cancer and Hodgkin's disease and low TB prevalence in patients with cancers of the colon and genitourinary tract. Until recently, the specific association between urinary tuberculosis and urinary tract cancer had not been reported. A recently published nationwide cohort study in Taiwan showed that urinary tuberculosis is associated with the development of urothelial carcinoma (1.2% of patients with urinary TB versus 0.3% of nonurinary TB patients developed urothelial carcinoma, resp. (*P* < 0.001)). There was however no association with renal cell carcinoma [5].

Some authors have suggested the possibility of an etiological relationship between coexisting carcinoma and tuberculosis [6]. The etiological relationship may be explained by chronic inflammatory mucosal damage initiating a sequence of metaplasia and dysplasia resulting in neoplastic change [6], as seen in cases of colon cancer in which ulcerative lesions of intestinal tuberculosis are precursors of carcinomas [7]. Another possibility for the coincidence of TB and malignancy is that cancer-associated altered host immunity can increase susceptibility to active tubercular infection [4]. Occurrence of both diseases at the same site (same kidney) may be explained by invasion of a dormant tubercular lesion by carcinoma causing activation and endogenous reinfection [4]. Locally produced tumor peptides or antigens may also upset the milieu of a granuloma and allow the TB organisms to proliferate. It is impossible to say which disease preceded the other in our patient.

This case highlights several challenges in the diagnosis and management of rare tumors such as collecting duct tumors, which are known to be aggressive with no established standard approach to therapy. Systemic chemotherapy with carboplatin/paclitaxel was chosen for our patient based on previous reports of response of collecting duct carcinoma to this chemotherapy combination [8]. Our patient however had disease progression on this regimen. By the time of progression, pathology review with adjunctive immunostaining tests became available and confirmed high-grade urothelial carcinoma. Gemcitabine/cisplatin has been established as a standard therapy for urothelial malignancies with favorable risk-benefit ratio over older cisplatin-containing regimens [9] and response rates ranging from 43.6 to 49% [9, 10]. Although our patient did not have a response to gemcitabine/cisplatin, the case highlights the value of adjunctive immunostains in the evaluation of rare histological types, as this may lead to changes in management.

A microbiologic diagnosis of TB is usually made by isolation of the causative organism from urine or biopsy material on conventional solid media or by an automated system such as radiometry. In recent years, nucleic-acid amplification techniques, such as PCR, have been investigated extensively for the detection of* M. tuberculosis* and other mycobacteria in clinical specimens, notably sputum. Relatively few studies have specifically evaluated PCR for detection of genitourinary TB, and these show the technique to be sensitive and specific [11].

This case illustrates that while the concomitant occurrence of renal tuberculosis and renal tumors is rare, the likelihood of concurrence should be kept in mind especially in patients from tuberculosis endemic areas and in patients with equivocal symptoms [6].
